# Translational research platforms integrating clinical and omics data: a review of publicly available solutions

**DOI:** 10.1093/bib/bbu006

**Published:** 2014-03-07

**Authors:** Vincent Canuel, Bastien Rance, Paul Avillach, Patrice Degoulet, Anita Burgun

**Keywords:** translational medical research, biomedical research, clinical data, high-throughput technologies, information storage and retrieval

## Abstract

The rise of personalized medicine and the availability of high-throughput molecular analyses in the context of clinical care have increased the need for adequate tools for translational researchers to manage and explore these data. We reviewed the biomedical literature for translational platforms allowing the management and exploration of clinical and omics data, and identified seven platforms: BRISK, caTRIP, cBio Cancer Portal, G-DOC, iCOD, iDASH and tranSMART. We analyzed these platforms along seven major axes. (1) The community axis regrouped information regarding initiators and funders of the project, as well as availability status and references. (2) We regrouped under the information content axis the nature of the clinical and omics data handled by each system. (3) The privacy management environment axis encompassed functionalities allowing control over data privacy. (4) In the analysis support axis, we detailed the analytical and statistical tools provided by the platforms. We also explored (5) interoperability support and (6) system requirements. The final axis (7) platform support listed the availability of documentation and installation procedures. A large heterogeneity was observed in regard to the capability to manage phenotype information in addition to omics data, their security and interoperability features. The analytical and visualization features strongly depend on the considered platform. Similarly, the availability of the systems is variable. This review aims at providing the reader with the background to choose the platform best suited to their needs. To conclude, we discuss the desiderata for optimal translational research platforms, in terms of privacy, interoperability and technical features.

## INTRODUCTION

Personalized medicine aims at establishing links between biomolecular characterizations, patient conditions, treatment effectiveness and adverse effects, and thus providing patients with the best individual treatment [1]. Most of the advances on personalized medicine have been made possible by breakthrough improvements of biomolecular knowledge and technologies over the past decade. During that period, many high-throughput technologies have been developed to investigate various aspects of cellular processes, such as sequence and structural variations of the genome, transcriptome, epigenome, proteome and interactome (all these data are colloquially called omics data). Several recent reviews have provided in-depth discussion of some of these technologies [2–8]. Integrative genomics and systems biology, driven by this new knowledge and technologies, have greatly advanced our understanding of human diseases [9]. For example, there has been new evidence regarding the metastatic colon cancer that mutations activating the KRAS gene abrogate the therapeutic effect of anti-Epidermal Growth Factor Receptor therapies (like Cetuximab-*ERBITUX*®, or Panitumumab-*VECTIBIX*) [10–12].

A considerable and growing amount of omics data is generated by these high-throughput technologies, covering a broad spectrum of domains. These omics data need to be considered in the context of the cellular processes to achieve their full potential. With >1500 different biomolecular-related databases listed in the latest Nucleic Acids Research database issue [13], researchers need tools to find the relevant information in the maze of biological data available. Several systems have been developed to address this need and help scientists work with omics data, e.g. Gene Expression Omnibus [14], Array Express [15] or PRIDE [16]. However, omics data also have to be analyzed together with clinical data to be useful for translational researchers and beneficial to patients. Such systems do not provide a solution to the clinical and omics data integration problem.

Clinical data warehouses (CDWs) are now largely used to integrate data from a variety of clinical sources (e.g. biology results, imaging) and present a unified view on clinical data. They provide a valuable resource for many cases, e.g. to identify a population with common characteristics and to discover significant associations among phenotypes [17]. Many CDW implementations rely on the ‘Informatics for Integrating Biology and the Bedside’ (i2b2) infrastructure [18], an NIH-funded National Center for Biomedical Computing based at Partners HealthCare System in Boston, which has been adopted by numerous academic hospitals around the world [19,20]. CDWs are the precondition for integrating clinical and omics data. They need to be suitably extended to handle molecular information. As our understanding of diseases becomes ever more stratified by their genomic signatures, larger data sets will be needed to establish diagnosis and treatment protocols. A data network that integrates research data on the molecular makeup of diseases with clinical data on individual patients could drive the development of a more accurate classification of disease and ultimately enhance diagnosis and treatment [21]. This can only be achieved through large federated pools of information that include patient genomic data and their health histories.

In recent years, new trends in clinical and omics data management and analysis have emerged. Several options have been taken to produce solutions regarding ‘informatics methods that connect molecular entities to clinical entities’ [22]. Among them, translational research platforms, able to integrate large data sets of clinical information with omics data, are now actively being developed.

Such translational research platforms should be able to blend in researchers’ workflow for an optimal use. Therefore, they should provide (i) the storage and integration of clinical and omics data; (ii) an analysis framework, enabling scientists to explore their data and generate hypotheses; and (iii) additional information cross-referenced from external databases (e.g. link to a specific gene description in published literature or public databases).

In this review, we focused on the main storage, integration and analysis platforms for translational research. Our goal is to provide translational researchers with background knowledge to approach the main translational research platforms currently available. We describe core functionalities, main features and limits of each platform, based on the published information.

## MATERIALS AND METHODS

We used PubMed® to explore the scientific literature and subsequently identified 2359 articles potentially describing translational platforms (PubMed® queries are available in Appendix 1). We manually reviewed the articles to identify systems (i) enabling the integration of private clinical and omics data and (ii) providing researchers with data analysis functionalities. Analysis of the accessed publications was completed by Google® search and analysis of the paper references to search for other possible candidate solutions. Seven of the main translational research platforms were included in the review: BRISK [23], caTRIP [24], cBio Cancer Genomics Portal for Cancer Genomics [25], Georgetown Database of Cancer (G-DOC) [26], integrated clinical omics database (iCOD) [27], integrating data for analysis, anonymization and sharing (iDASH) [28] and tranSMART [29]. We did not include commercial products in our review.

Using publicly available resources (i.e. original articles published in PubMed® before 15 September 2013 describing the systems and dedicated websites), we identified the main features of each platform. These features were analyzed along seven major axes. (1) The *‘*community’ axis regrouped information regarding initiators and funders of the project, as well as availability status and references. (2) We regrouped under the ‘information content’ axis the nature of the clinical and omics data handled by each system. (3) The ‘privacy management environment’ axis encompassed functionalities allowing control over data privacy. (4) In the ‘analysis support’ axis, we detailed analytical and statistical tools provided by the platforms. We also explored (5) ‘interoperability support’ and (6) ‘system requirements’. The final axis (7) ‘platform support’ listed the existence of documentation and installation procedures.

We also directly contacted the authors of the original papers and asked them to assert our findings. Four platforms (of the seven) have responded, namely, BRISK, cBio Cancer Genomics Portal, iDASH and tranSMART.

## RESULTS

In this section, we describe the basic functionalities available for each platform. Platforms features, technical description of the systems and a snapshot of their analytical functions are provided in Table 1.

**Table 1:** Details of the main features for the seven included platforms

Platform	tranSMART	cBioPortal	BRISK	iDASH	iCOD	G-DOC	caTRIP
Community							
Institution/project initiators	Johnson & Johnson, USA	Memorial Sloan-Kettering Cancer Center, NY, USA	University of British Columbia, Canada	iDASH team, USA	Tokyo Medical Dental University, Japan	Georgetown University, DC, USA	caBIG consortium, USA
Funding	Initially Johnson & Johnson funded—now public-private consortia	NIH and industry grants	Public and private consortium	NIH	Public	HSS	NIH
Reference	Szalma *et al.* 2010	Cerami *et al.* 2012	Tan *et al.* 2011	Ohno-Machado *et al.* 2012	Shimokawa *et al.* 2010	Madhavan *et al.* 2011	McConnell *et al.* 2008
PMID	20642836	22588877	21712248	22081224	21143802	21969811	19108734
Software availability	Open source	Free for academic use, commercial licenses available	Open source	Open source	Not distributed	Not distributed	Not distributed
Licensing	GPL v3	Free for academic use, commercial licenses available	GPL v2	BSD-like	Unknown	Proprietary	Open source
User mailing list or support	Yes	Yes	No	Yes	Unknown	Yes	No
URL	http://transmartfoundation.org	http://www.cbioportal.org/	http://genapha.icapture.ubc.ca/brisk/index.do	http://idash.ucsd.edu	http://omics.tmd.ac.jp/icod/	https://gdoc.georgetown.edu/gdoc/	https://cabig-stage.nci.nih.gov/community/tools/caTRIP/
Information content							
Clinical data							
Demographics	Yes	Yes	Yes	Yes	Yes	Yes	Yes
Outcomes	Yes	Yes	No^a^	Yes	Yes	Yes	Yes
Biological results	Yes	Yes	Yes	Yes	Yes	Yes	Yes
Images	No	No^a^	No	Yes	Yes	No	Unknown
Structured clinical research data	Yes	Yes	Yes	Yes	Yes	Yes	Yes
‘omics’ data							
mRNA expression	Yes	Yes	No	Yes	Yes	Yes	Unknown
miRNA expression	No	Yes	No	Yes	No	Yes	Unknown
SNPs	Yes	Yes	Yes	Yes	No	Yes	Unknown
Copy number variations	Yes	Yes	Yes	Unknown	No	Yes	Unknown
DNA methylation	No	Yes	Yes	Unknown	No	Yes	Unknown
Protein/phosphoprotein expression	Yes	Yes	No	Unknown	No	Yes	Unknown
Structural rearrangements	No	Yes	No	Unknown	No	No	Unknown
Privacy management environment							
Anonymization/de-anonymization	No	No	No	Yes	No	No	Yes
Analysis supports							
Statistical framework	R	R, Matlab	*In-house components*	R	R	R	*In-house components*
Analytical features	GenePattern, Bioconductor Plink	*In-house components*	*In-house components*	*Privavy protecting analytical features*	*In-house components*	Bioconductor	*In-house components*
Visualization tools	Haploview, IGV and output of analytical tools	*In-house components*	*In-house components*	*In-house components*	*In-house components*	Java TreeView, Jbrowse, Cytoscape	*In-house components*
Interoperability support							
Ontologies/standard terminologies	i2b2 ontology features	No	No	Yes	No	No	Yes
Collaborative environment	Yes	Yes	Yes	Yes	Unknown	Yes	Yes
Secure environment	Yes	Yes	Yes	Yes	Unknown	Yes	Yes
Support of multisite requests	No	No	No	Yes	No	No	Yes
APIs/web services interface	JBoss	Yes	Unknown	Yes	Unknown	Unknown	Yes
System requirements							
Operating system	Linux	Linux	Linux	Linux	Unknown	Linux	Unknown
Database management system	Oracle 11/PostgreSQL	MySQL	MySQL	Multiple	PostgreSQL	Oracle	Oracle, MySQL
Database type	Centralized	Centralized	Local + external	Per application/Web service	Centralized	Centralized	Distributed
Software dependencies	SOLR, R, i2b2	None	None	MIDAS, varies per application	R	Adobe Flex framework, Java TreeView, Cytoscape, Jbrowse, Lucene	caGRID tools
Main programming language	Java/GRAILS	Java, Javascript	Java	Multiple	Java	GRAILS	Java
Server side	Tomcat/JBoss	Tomcat	Tomcat	Multiple	Tomcat	Unknown	Unknown
Client-side interface	Web browser	Web browser	Web browser	Web browser	Unknown	Web browser	Java
Platform support							
Installation procedures	Yes	Yes	Yes	Per application/web service	Not available	N/A	Not available
Configuration documentation	Yes	Yes	Yes	Wiki per application	Not available	N/A	Not available
User documentation	Yes	Yes	Yes	Wiki per application	Not available	yes	Not available

^a^Marks a disagreement between our analysis and the platforms authors. The table indicates the result of our analysis.

### Overview of translational platforms

#### BRISK: Biology-Related Information Storage Kit (2011)

The Biology-Related Information Storage Kit (BRISK) is a package of three open-source web-based applications providing a cohesive data integration and management platform. It was initially developed to provide a data-sharing solution for researchers in the AllerGen (The Allergy, Genes and Environment Network) consortium (http://www.allergen-nce.ca). BRISK can handle clinical phenotype description and somatic mutation (single-nucleotide polymorphisms) information. It provides researchers with genome-wide association studies (GWAS) analysis capabilities. This solution also includes a laboratory-oriented application managing physical sample, subject and container data.

#### caTRIP (2006)

The caTRIP platform was developed as a component of the caBIG project in the early 2000s to allow users to query across the caBIG grid. The caBIG was a U.S. National Cancer Institute program. Its goal was to develop an open-source network across the United States for secure exchanges on cancer research. The goals of caTRIP include allowing physicians to find patients with similar profiles, analyze their outcomes and find information about successful treatments across the caBIG data grid. The system interoperates with several caBIG applications, including the Tumor Registry, a clinical system used to collect data; the cancer Text Information Extraction System, a natural language processing (NLP) tool designed to extract clinical knowledge from surgical pathology free-text report using controlled terminologies; the caTissue CORE, a tissue bank repository; the Cancer Annotation Engine and the caIntegrator, a tool for storing, querying and analyzing data.

#### cBio Cancer Genomics Portal (2012)

Developed at Memorial Sloan-Kettering Cancer Center (MSKCC), the cBio Cancer Genomics Portal is an open-source platform designed to facilitate the access of translational researchers to data sets generated by large-scale cancer genomics projects, like The Cancer Genome Atlas (http://cancergenome.nih.gov/) and the International Cancer Genome Consortium (http://icgc.org/). It integrates de-identified clinical data, such as phenotype description, survival or disease-free survival intervals, with major high-throughput omics data (DNA, messenger RNA - mNRA, and proteins). Additionally, pathology images can be accessed through embedded TGCA cancer digital slide archive visualization (http://cancer.digitalslidearchive.net/). Images can be accessed through embedded TGCA cancer digital slide archive visualization (http://cancer.digitalslidearchive.net/). Advanced visualization, analysis and export functionalities are provided. The public online version mainly stores published large-scale cancer genomics data sets, while a private instance of the portal can be set up locally by research groups willing to import their own research data sets.

#### G-DOC Georgetown Database of Cancer (2012)

Developed at the Lombardi Comprehensive Cancer Center at Georgetown University, the Georgetown Database of Cancer (G-DOC) is a translational informatics infrastructure aiming to facilitate translational and systems-based medicine. It was designed specifically to address the activation barrier for use of biomedical informatics tools by basic, clinical and translational researchers. G-DOC integrates patient characteristics (e.g. demographics, structured clinical research data) and clinical outcomes data with four major high-throughput omics data (DNA, mRNA, microRNA and metabolites) in a unified environment. The associated framework, the Georgetown Clinical and Omics Development Engine [30] (G-CODE) contains a wide array of bioinformatics and systems biology tools dedicated to data analysis and visualization.

#### iCOD: Integrated Clinical Omics Database (2010)

The Integrated Clinical Omics Database (iCOD) was developed to combine comprehensive clinicopathological and molecular information of patients to provide a holistic understanding of the diseases. iCOD can handle omics data like gene expression profiles and heterogeneous clinical information such as detailed phenotypes, radiology images or laboratory test results. Locally developed integrated view maps of diseases are provided to summarize the interrelation of clinical and omics data and represent plausible disease pathways.

#### iDASH: Integrating data for analysis, anonymization and sharing (2011)

iDASH is a National Center for Biomedical Computing. iDASH provides researchers all over the United States with a powerful computational infrastructure required for data integration and data analysis. iDASH also distributes tools and algorithms, focused on sharing data in a privacy-preserving manner. iDASH provides biomedical and behavioral researchers with access to data, software and a high-performance computing environment, thus enabling them to generate and test new hypotheses.

#### tranSMART (2010)

This platform was initially developed as a precompetitive collaboration platform for pharmaceutical firms by a private consortium before being released in the open-source community (the tranSMART Foundation is now in charge of the sustainability and code development). The platform is based on the open-source i2b2 CDW [18]. It is built to help scientists develop and refine research hypotheses by investigating correlations between phenotypic and omics data. TranSMART can handle structured data from clinical trials (demographics, outcomes, laboratory results and clinical phenotypes) and aligned high-content biomarker data such as gene expression profiles, genotypes, metabolomics and proteomics data. It provides researchers with analysis tools able to generate advanced descriptive and analytics statistics.

## COMPARISON OF THE TRANSLATIONAL RESEARCH PLATFORMS

In this section, we describe and compare the features and architecture choices of the translational platforms. The information presented was that available to us in December 2013.

### Information content

#### Clinical data

The term ‘clinical data’ encompass a wide array of data: demographics characteristics (e.g. age, sex and ethnicity), physical examinations, patient history, medical diagnoses (using standard terminologies, including ICD10 codes), treatments, laboratory test results (e.g. from standard blood test to advanced bio-molecular determination), pathology reports in free text, radiology images, clinical outcomes (e.g. survival rates) and so forth. Capturing and managing such highly complex data, for every patient, is itself a challenging issue for bioinformaticians and researchers alike. Moreover, the provenance of the information is diverse. Clinical ‘care’ data are often stored in electronic health records (EHR) or CDW, whereas clinical ‘research’ data are collected in electronic case report forms or clinical data management systems. Consequently, the management of the data needs to be adapted (including for the modeling aspects, formatting of the data and Extract, Transform, Load (ETL) processes).

BRISK and the cBio Cancer Genomics Portal focus mainly on the exploration of omics data. In these platforms, clinical data are collected and stored to enable sample categorization and to perform specific analysis (e.g. type of pathology for a GWAS analysis in BRISK and disease-free intervals for a survival analysis in the cBio Cancer Genomics Portal). caTRIP, G-DOC, iCOD, iDASH and tranSMART also focus on the exploration of clinical data. iDASH provides numerous NLP and image analysis tools, and manages the documents using MIDAS (http://midasplatform.org/), an open-source solution. In tranSMART, phenotypic data are stored using the i2b2 data model consisting of an entity attribute value pair-derived star-schema [18]; G-DOC and iCOD use their own database format.

#### Omics data

Regarding omics data, each platform supported a specific set of data, depending on the initial aims of the platform and the needs of the researchers driving the project. G-DOC supports four types of omics data: mRNA, microRNA, copy number variation and metabolite mass spectrometry. As a translational research platform initially aimed at the drug development field, tranSMART supports multiple omics data sets useful to pharmaceutical companies: gene expression profiles, genotypes, serum protein panels, metabolomics and proteomics data. The BRISK platform is focused on GWAS association study: single-nucleotide polymorphisms are the only omics data supported. The cBio Cancer Genomics Portal is able to support a wide range of omics data set produced by large-scale studies: mutation data, copy number alterations, microarray-based and RNA sequencing-based mRNA expression changes, DNA methylation values and protein and phosphoprotein levels. iCOD includes molecular omics data such as comparative genomic hybridization and gene expression profiles.

### Interoperability support

Most of the platforms do not provide support for standard terminologies and ontologies. Only iDASH and caTRIP were built to natively support a limited set of terminologies. TranSMART presently handles the use of terminologies (e.g. ICD10 or LOINC). Simple mappings can be managed through the i2b2 functionalities [31,32].

A collaborative and secure environment is also provided by every platform except iCOD (information not available). This enables researchers to securely share and work concurrently on stored data sets, potentially speeding up the research process.

Surprisingly, none of the platforms can fully be integrated in a global framework: standard formats such as CDISC ODM [33] or HL7 CDA [34] are not handled as entry format, and outputs are not always compatible with existing bioinformatics analysis pipelines.

### Analysis support features

#### Visualization, statistical and analytical tools

Analytical features provided by the cBio Cancer Genomics Portal, G-DOC, iCOD, iDASH and tranSMART mainly rely on a third-party tool, like the *R* statistical software, directly embedded into the platforms. They provide ready-to-use analytical scripts implementing the main tests and analytical tools used by the researchers (including but not limited to *t*-test and principal component analysis). These analytical scripts are made available through user-friendly graphical interfaces. Therefore, the end-user does not need advanced computational or scripting knowledge to be able to leverage the analysis features. For many aspects of the omics analysis framework, tranSMART leverages Bioconductor [35] and GenePattern [36] (a system provided by the Broad Institutes), while G-DOC and iCOD use mainly tools developed in-house. The analysis tools used by BRISK are not stated. We will not detail the types of analysis available, as it is highly dependent on clinical and omics data managed by the systems and might therefore evolve at a fast pace.

On top of the analysis framework, multiple visualization tools are provided. This type of tools included third-party software (e.g. the Integrative Genome Viewer) and in-house components, such as ‘OncoPrint’ for the cBio Cancer Genomics Portal platform and the ‘Web Information Service’ in BRISK. G-DOC leverages the open-source (and widely used tool) Cytoscape [37] to display interaction network, Java TreeView for heatmaps as well as several in-house components. Metacore™ (http://thomsonreuters.com/metacore/) from Thomson Reuters® is available in tranSMART. These kinds of visualization tools are crucial features for a translational research platform, as they ease the interpretation of complex analysis results.

In addition to the analytical tools, most of the systems implement export functionalities compatible with SAS®, *R* or MS Excel® software, allowing for advanced analysis by statistician experts. To further facilitate the results interpretation, platforms added additional contextual information compiled from public sources. For example, mapping tools were implemented into BRISK to access contextual information from external databases, such as the Kyoto Encyclopedia of Genes and Genomes (KEGG) [38]. Similar tools were implemented in all the platforms.

### Security and privacy tools

#### Data privacy

Handling patient-level clinical research data is a highly sensitive issue, regarding ethics and privacy aspects. Unsurprisingly, all the platforms included basic security policies. Unauthorized access to data sets is prevented by a user authentication process (personal credentials for each researcher), combined with specific access rights. iDASH has specifically been designed to handle the challenge of privacy. The system proposes NLP tools for de-identification, as well as numerous statistical tools adapted to preserve patient privacy. In the other systems, the de-identification steps have to be performed either before loading the data in the system or, when needed, during the export process.

### Platform support

#### Platform documentation

Most of the platforms described in this review are still at early stages of their development and lack adequate documentation. However, active user communities are providing valuable technical help both for installation and use of the systems. Ready-to-use platforms (e.g. G-DOC or the cBio Cancer Genomics Portal) provide tutorials or training for their users.

#### Installation and management of the platform

The choice of a platform strongly depends on various considerations: goals, resources and also from practical aspects guided by ethical and legal requirements. Some platforms provide data storage and analysis ‘as a service’ for translational researchers. For example, G-DOC is not open-source software, and its code is not publicly available. However, research groups may use the system by signing an agreement. In this case, researchers’ data have to be shared with Georgetown University and will be stored on G-DOC’s servers. The platform is fully functional and can be used directly by the user after loading data. Deployment issues are cut to the absolute minimum, as installation and management are carried out by G-DOC’s team. The online version of the cBio Cancer Genomics Portal is based on the same principle. BRISK, iDASH, the local version of the cBio Cancer Genomics Portal and tranSMART are deployed ‘on-site’ and therefore require important infrastructures (e.g. web and Java servlet servers, databases) that are probably out-of-reach for the average translational clinician or researcher. Noticeable efforts have been made to ease the installation and the use of such complex systems. For example, tranSMART provides a ready-to-use version through tranSMART virtual appliance [39].

## DISCUSSION

### Current lesson from platform comparison

The simultaneous reduction of the cost of high-throughput technologies and the dissemination of EHR lead to an exponential increase of the amount of omics and clinical data made available for researchers. The exploration of such amount of data requires specific tools and methods that are complex to deploy. Multiple translational research platforms have been developed to answer these new needs of exploration and analysis capabilities, together with a relative simplicity of deployment. For research groups, the selection of an adequate platform might be a difficult task due to the heterogeneity of their features. Moreover, most of the systems have been published in a short period of time. It is worth noting that the various publications describing the systems discussed in this review do not cite other systems as related work, and consequently do not propose a comparison of features. This review provides an overview of non-commercial solutions available and their main features. To the best of our knowledge, this review is the first study comparing translational research platforms.

### Related works

In this review, we described the main platforms providing both integration and analysis features for clinical and omics data. Platforms not updated in recent years were not considered for this review. We detailed the functionalities of caTRIP despite its lack of recent updates owing to its precursor status in the field of translational research.

Many approaches have been developed to answer similar problems on limited data sets (e.g. often to study specific research questions). We decided to include in this review only generic solutions; consequently, we have not detailed specialized systems.

Several translational platforms handling clinical data have been developed over the past decade (e.g. i2b2, STRIDE [40] - Stanford University, BTRIS [41]). In part due to its architecture, i2b2 is widely used across the world for ‘on-site’ translational research platforms. I2b2 is composed of a series of software modules called cells that are interconnected through web services. Cells share a common messaging protocol and can be developed by different groups. This specificity allows a large variety of usage of the platform, as well as the development of components dedicated to specific needs. For example, the Eureka! system extends the capacities of i2b2 to be able to handle temporality in phenotypes, and the ONCO-i2b2 [32] and BioSTOR [42] initiatives aim at providing an i2b2 platform with biobank analysis features. Several of the platforms reviewed, including BRISK, iDASH and tranSMART, have adopted web service-based architecture.

### Desiderata for translational research platforms

#### Privacy

Several of the platforms included in this study were not available for local deployment. Instead, they use a client/server architecture, for which the server is not controlled by the final user. This could lead to potential problems regarding data privacy regulations. Storing and sharing clinical and omics data are very sensitive topics in the translational research field, as they raise both ethics and privacy issues. Clinical research groups are often bound to stringent privacy rules (e.g. the Health Insurance Portability and Accountability Act in the United States; the Data Processing, Data Files and Individual Liberties Act in France). The use of remote (including cloud-based) solutions is still debated. While they open tremendous possibilities, especially regarding cost-efficiency, computing power and flexibility, a cloud-based platform should not be used until privacy and data-sharing issues have been carefully evaluated. The same rules should apply for privately or publicly owned remote platforms. Arguably, integration solutions do not only need to enable storage and exploration of the data, but also to make these functionalities available in a controlled environment compatible with government regulations and good practices. Consequently, privacy issues will often require the ability to install translational research platforms within the institution’s boundaries.

#### Interoperability and standards

Among the other goals, translational platforms claim (1) to enable efficient data sharing, for example, to increase the quantity of data available for rare diseases and (2) to ease data integration. Both goals need interoperability and comparability of the data. However, none of the platforms described in this review were able to interoperate directly with EHR or personal health records. The systems are not able to natively import data in international exchange standards such as HL7 CDA [34] or CDISC ODM [33]. Efficient data integration also requires that translational research platforms can be blended into existing data collection processes within the institutions. Platforms should provide reusable ETL pipelines to handle not only research data (e.g. text or spreadsheet files) but also standard clinical and omics messaging format. The systems reviewed in that study all presented simple ways to export data for further analysis, and efforts to integrate outputs with a bioinformatics analytical framework are ongoing.

Most of the platforms explored in this review have adopted modular structures, which allow—to some extent—the connection to classic analytical tools (e.g. Plink or GenePattern for tranSMART). However, platform modules are not often designed to be shared easily with other platforms. The increased development of customizable and reusable tools and libraries would be a great help for the field. Similarly, the adoption of APIs has not yet reached its full potential and would allow such customizable connections, for example, by enabling simple access to the data or easy setup of analytical workflows.

Moreover, the use of standard terminologies and ontologies is another key component of interoperability and data sharing. Surprisingly, the reviewed platforms offer limited ability to handle such features. We claim that translational platforms have to be able to manage local alignment to controlled vocabularies. In addition, the use of international standard terminologies (e.g. ICD-10, SNOMED CT) would allow using the subsumption properties and the semantic links in terminologies, thus enabling computer reasoning.

#### Heterogeneity of granularity of the data models

Integration of omics and clinical research data, and clinical care data might lead to discrepancies in the representation of data. More specifically, clinical research data collection is constrained (with respect to a protocol, enabling proper comparability within the study), and omics data are stored/produced in a standardized format (e.g. MIAME), whereas clinical care data are collected whenever needed for the care of patients. Most of the systems presented in this study use a representation based on a clinical research data model. Consequently, the integration of care data in the systems requires transformation not only of the format but of the model as well. Clinical research and care data are difficult to handle, partially due to their heterogeneous nature and also due to temporal issues. It should be noted that none of the platforms included in this review were currently able to manipulate complex temporal data (e.g. medication intervals), leaving room for improvement.

#### Deployment and maintenance

Although most of the ‘on-site’ platforms described in this review are mature projects and already provide translational researchers with advanced capabilities, we have to make clear that the deployment and maintenance of these platforms require the assistance of an IT team, as an adequate computer and network infrastructure is needed.

Most of the platforms embed ready-to-use analytical tools and visualization. The addition of new features, eased by the modular architecture of the systems, may require development by statistics or computer experts. However, for important features, the systems often leverage widely adopted solutions (e.g. the *R* statistical software and the i2b2 CDW model). This provides the benefit of an active community of developers and users within the translational research field and also contributions from other fields.

#### Closing the loop

The ultimate goal of translational medicine is enabling personalized care. Nowadays efforts are made to populate translational research platforms with patient data to fuel discovery. Allowing real-time data-driven decision algorithms to leverage translation research results in the context of clinical care [43] should be a short-term objective.

## CONCLUSION

The rise of personalized medicine together with the reduction of the cost of omics technologies has opened fantastic opportunities for primary health care physicians to include genomics consideration in the treatment of patients. However, the explosion of data available leads to the need for architecture allowing the exploration and management of combination of omics and clinical data for translational research.

In this review, we explore seven translational research platforms (BRISK, caTRIP, cBio Cancer Genomics Portal, G-DOC, iCOD, iDASH and tranSMART) and compare their features. We detailed several aspects of the platforms. For each platform, we listed the types of clinical and omics data handled. We compared the exploration, analysis and visualization tools provided, as well as the nature of these tools. Privacy being a crucial issue, we also explored the systems with respect to this question. Finally, we considered the practical issues of deployment and maintenance. Despite a tremendous amount of work and numerous features, the systems available at the time of this review still have room for improvement. We discussed the desiderata for enhanced translation research platforms especially in terms of data exchange and interoperability, as well as data privacy.

**Key Points**

- Personalized medicine aims at establishing links between biomolecular characterizations, patient conditions, treatment effectiveness and adverse effects, and thus providing patients with the best individual treatment.
- The rise of personalized medicine and the availability of high-throughput molecular analyses in the context of clinical care have increased the need for adequate tools for translational researchers to manage and explore these data.
- We reviewed the biomedical literature for translational platforms allowing the management and exploration of clinical and omics data, and identified seven platforms: BRISK, caTRIP, cBio Cancer Portal, G-DOC, iCOD, iDASH and tranSMART.
- We analyzed these platforms along seven major axes: community, information content, privacy management environment, analysis support, interoperability support, system requirements and platform support.
- We observed a large heterogeneity regarding the capability to phenotype information in addition to omics data, their security and interoperability features, and discussed the desiderata for optimal translational research platforms, in terms of privacy, interoperability and technical features.

## Supplementary Material

Supplementary Data

## APPENDIX 1

**Table:** Detailed queries used for the interrogation of PubMed® database (queries last run on 21 October 2013).

Query number	Query	Items found
Search using MeSH terms, covering the past 5 years :		
1	“Computational Biology”[Majr] OR “Translational medical research”[Majr] OR “Biomedical research”[Majr:NoExp] OR (“Medical Informatics”[Majr] NOT (“Decision Making, Computer-Assisted”[Mesh] OR “Decision Support Techniques”[Mesh]))	153 062
2	“Information storage and retrieval”[Mesh] OR “data repository”[Title/Abstract] OR “data repositories”[Title/Abstract] OR “data base”[Title/abstract] OR “data bases”[Title/Abstract] OR “database”[Title/Abstract] OR “databases”[Title/Abstract] OR “platforms”[Title/Abstract] OR “platform”[Title/Abstract] OR “warehouse”[Title/Abstract] OR “warehouses”[Title/Abstract]	304 152
3	“clinical”[Title/Abstract] OR “medical”[Title/Abstract] OR “biomedical”[Title/Abstract] OR “translational”[Title/Abstract]	2 862 543
4	“omics”[Title/Abstract] OR “genomics”[Title/Abstract] OR “transcriptomics”[Title/Abstract] OR “proteomics”[Title/Abstract] OR “metabolomics”[Title/Abstract] OR “biomarker”[Title/Abstract] OR “biomarkers”[Title/Abstract] OR “molecular”[Title/Abstract] OR “biological”[Title/Abstract]	1 327 752
5	“Archaea”[Mesh] OR “Bacteria”[Mesh] OR “Organism forms”[Mesh] OR “Viruses”[Mesh] OR “Eukaryota”[Mesh] NOT “Humans”[Mesh]	4 779 281
6	(#1 AND #2 AND #3 AND #4) NOT #5	1785
7	**Filters: published in the last 5 years**	**1119**
Search without MeSH terms, covering the past 1 year:		
8	“Information storage and retrieval”[Mesh] OR “data repository”[Title/Abstract] OR “data repositories”[Title/Abstract] OR “data base”[Title/abstract] OR “data bases”[Title/Abstract] OR “database”[Title/Abstract] OR “databases”[Title/Abstract] OR “platforms”[Title/Abstract] OR “platform”[Title/Abstract] OR “warehouse”[Title/Abstract] OR “warehouses”[Title/Abstract]	304 152
9	“clinical”[Title/Abstract] OR “medical”[Title/Abstract] OR “biomedical”[Title/Abstract] OR “translational”[Title/Abstract]	2 862 543
10	“omics”[Title/Abstract] OR “genomics”[Title/Abstract] OR “transcriptomics”[Title/Abstract] OR “proteomics”[Title/Abstract] OR “metabolomics”[Title/Abstract] OR “biomarker”[Title/Abstract] OR “biomarkers”[Title/Abstract] OR “molecular”[Title/Abstract] OR “biological”[Title/Abstract]	1 327 752
11	(#1 AND #2 AND #3)	8914
12	**Filters: published between 2012/09/19–2013/10/21**	**1432**
13	**(#7 OR #12) AND english[Language]**	**2359**

## References

[1] Hood L, Flores M (2012). A personal view on systems medicine and the emergence of proactive P4 medicine: predictive, preventive, personalized and participatory. N Biotechnol.

[2] Licatalosi DD, Darnell RB (2010). RNA processing and its regulation: global insights into biological networks. Nat Rev Genet.

[3] Wang Z, Gerstein M, Snyder M (2009). RNA-Seq: a revolutionary tool for transcriptomics. Nat Rev Genet.

[4] Farnham PJ (2009). Insights from genomic profiling of transcription factors. Nat Rev Genet.

[5] Park PJ (2009). ChIP-seq: advantages and challenges of a maturing technology. Nat Rev Genet.

[6] Metzker ML (2010). Sequencing technologies—the next generation. Nat Rev Genet.

[7] Beyer A, Bandyopadhyay S, Ideker T (2007). Integrating physical and genetic maps: from genomes to interaction networks. Nat Rev Genet.

[8] Laird PW (2010). Principles and challenges of genomewide DNA methylation analysis. Nat Rev Genet.

[9] Hawkins RD, Hon GC, Ren B (2010). Next-generation genomics: an integrative approach. Nat Rev Genet.

[10] Jung Y, Lee S, Choi HS (2011). Clinical validation of colorectal cancer biomarkers identified from bioinformatics analysis of public expression data. Clin Cancer Res.

[11] De Roock W, Claes B, Bernasconi D (2010). Effects of KRAS, BRAF, NRAS, and PIK3CA mutations on the efficacy of cetuximab plus chemotherapy in chemotherapy-refractory metastatic colorectal cancer: a retrospective consortium analysis. Lancet Oncol.

[12] Laurent-Puig P, Cayre A, Manceau G (2009). Analysis of PTEN, BRAF, and EGFR status in determining benefit from cetuximab therapy in wild-type KRAS metastatic colon cancer. J Clin Oncol.

[13] Fernandez-Suarez XM, Galperin MY (2012). The 2013 nucleic acids research database issue and the online molecular biology database collection. Nucleic Acids Res.

[14] Barrett T, Troup DB, Wilhite SE (2007). NCBI GEO: mining tens of millions of expression profiles—database and tools update. Nucleic Acids Res.

[15] Parkinson H, Sarkans U, Kolesnikov N (2011). ArrayExpress update—an archive of microarray and high-throughput sequencing-based functional genomics experiments. Nucleic Acids Res.

[16] Martens L, Hermjakob H, Jones P (2005). PRIDE: the proteomics identifications database. Proteomics.

[17] Tatonetti NP, Denny JC, Murphy SN (2011). Detecting drug interactions from adverse-event reports: interaction between paroxetine and pravastatin increases blood glucose levels. Clin Pharmacol Ther.

[18] Murphy SN, Weber G, Mendis M (2010). Serving the enterprise and beyond with informatics for integrating biology and the bedside (i2b2). J Am Med Inform Assoc.

[19] McMurry AJ, Murphy SN, MacFadden D (2013). SHRINE: enabling nationally scalable multi-site disease studies. Carter KW (ed. by). PLoS One.

[20] Zapletal E, Rodon N, Grabar N (2010). Methodology of integration of a clinical data warehouse with a clinical information system: the HEGP case. Stud Health Technol Inform.

[21] Committee on a Framework for Development of a New Taxonomy of Disease (2011). Toward Precision Medicine: Building a Knowledge Network for Biomedical Research and a New Taxonomy of Disease.

[22] Altman RB, Lewitter F, Kann M (2012). Introduction to translational bioinformatics collection. PLoS Comput Biol.

[23] Tan A, Tripp B, Daley D (2011). BRISK—research-oriented storage kit for biology-related data. Bioinformatics.

[24] McConnell P, Dash RC, Chilukuri R (2008). The cancer translational research informatics platform. BMC Med Inform Decis Mak.

[25] Cerami E, Gao J, Dogrusoz U (2012). The cBio cancer genomics portal: an open platform for exploring multidimensional cancer genomics data. Cancer Discov.

[26] Madhavan S, Gusev Y, Harris M (2011). G-DOC: a systems medicine platform for personalized oncology. Neoplasia.

[27] Shimokawa K, Mogushi K, Shoji S (2010). iCOD: an integrated clinical omics database based on the systems-pathology view of disease. BMC Genomics.

[28] Ohno-Machado L, Bafna V, Boxwala AA (2012). iDASH: integrating data for analysis, anonymization, and sharing. J Am Med Inform Assoc.

[29] Szalma S, Koka V, Khasanova T (2010). Effective knowledge management in translational medicine. J Transl Med.

[30] Madhavan S, Gusev Y, Harris MA (2011). G-CODE: enabling systems medicine through innovative informatics. Genome Biol.

[31] Wu ST, Liu H, Li D (2012). Unified Medical Language System term occurrences in clinical notes: a large-scale corpus analysis. J Am Med Inform Assoc.

[32] Segagni D, Tibollo V, Dagliati A (2011). The ONCO-I2b2 project: integrating biobank information and clinical data to support translational research in oncology. Stud Health Technol Inform.

[33] ODM Certification & Archiving and Interchange of Metadata http://www.cdisc.org/odm.

[34] Dolin RH, Alschuler L, Beebe C (2001). The HL7 clinical document architecture. J Am Med Inform Assoc.

[35] Zhang J, Carey V, Gentleman R (2003). An extensible application for assembling annotation for genomic data. Bioinformatics.

[36] Reich M, Liefeld T, Gould J (2006). GenePattern 2.0. Nat Genet.

[37] Shannon P, Markiel A, Ozier O (2003). Cytoscape: a software environment for integrated models of biomolecular interaction networks. Genome Res.

[38] Kanehisa M, Goto S (2000). KEGG: kyoto encyclopedia of genes and genomes. Nucleic Acids Res.

[39] TranSMART Wiki. tranSMART 1.1 Virtual Appliance https://wiki.transmartfoundation.org/display/TSMTGPL/tranSMART+1.1+Virtual+Appliance.

[40] Lowe HJ, Ferris TA, Hernandez PM (2009). STRIDE—An integrated standards-based translational research informatics platform. AMIA Annu Symp Proc.

[41] Cimino JJ, Ayres EJ (2010). The clinical research data repository of the US National Institutes of Health. Stud Health Technol Inform.

[42] Chakrabarty R, Tran T, Wolf W (2013). BioSTOR: developing an Institutional Biobank linked to the clinical record via i2b2. AMIA 2013 Summit on Clinical Research Informatics.

[43] Kohane IS, Drazen JM, Campion EW (2012). A glimpse of the next 100 years in medicine. N Engl J Med.

