# Supplementary material for: Translational research platforms integrating clinical and omics data: a review of publicly available solutions
Source: Brief Bioinform. 2014 Mar 7;16(2):280–90. doi: 10.1093/bib/bbu006 (PMC4364065; doi:10.1093/bib/bbu006)
Supplement: Supplementary Data [file supp_16_2_280__index.html]

Translational research platforms integrating clinical and omics data: a review of publicly available solutions — Translational research platforms integrating clinical and omics data: a review of publicly available solutions — Supplementary Data 

# Translational research platforms integrating clinical and omics data: a review of publicly available solutions

## Supplementary Data

files

**Files in this Data Supplement:**

- Supplementary Data - docx file
