# Supplementary material for: Translational research platforms integrating clinical and omics data: a review of publicly available solutions
Source: Brief Bioinform. 2014 Mar 7;16(2):280–90. doi: 10.1093/bib/bbu006 (PMC4364065; doi:10.1093/bib/bbu006)
Supplement: Supplementary Data [file supp_bbu006_20131021_BIB_Platforms_Appendix_BRE.docx]

**Appendix A**. Detailed queries used for the interrogation of PubMed® database (queries last run on October 21^st^, 2013).

**Search using MeSH terms, covering the last 5 years :**

| **Query number** | **Query** | **Items found** |
| --- | --- | --- |
| #1 | "Computational Biology"[Majr] OR "Translational medical research"[Majr] OR "Biomedical research"[Majr:NoExp] OR ("Medical Informatics"[Majr] NOT ("Decision Making, Computer-Assisted"[Mesh] OR "Decision Support Techniques"[Mesh])) | 153,062 |
| #2 | "Information storage and retrieval"[Mesh] OR "data repository"[Title/Abstract] OR "data repositories"[Title/Abstract] OR "data base"[Title/abstract] OR "data bases"[Title/Abstract] OR "database"[Title/Abstract] OR "databases"[Title/Abstract] OR "platforms"[Title/Abstract] OR "platform"[Title/Abstract] OR "warehouse"[Title/Abstract] OR "warehouses"[Title/Abstract] | 304,152 |
| #3 | "clinical"[Title/Abstract] OR "medical"[Title/Abstract] OR "biomedical"[Title/Abstract] OR "translational"[Title/Abstract] | 2,862,543 |
| #4 | "omics"[Title/Abstract] OR "genomics"[Title/Abstract] OR "transcriptomics"[Title/Abstract] OR "proteomics"[Title/Abstract] OR "metabolomics"[Title/Abstract] OR "biomarker"[Title/Abstract] OR "biomarkers"[Title/Abstract] OR "molecular"[Title/Abstract] OR "biological"[Title/Abstract] | 1,327,752 |
| #5 | "Archaea"[Mesh] OR "Bacteria"[Mesh] OR "Organism forms"[Mesh] OR "Viruses"[Mesh] OR "Eukaryota"[Mesh] NOT "Humans"[Mesh] | 4,779,281 |
| #6 | (#1 AND #2 AND #3 AND #4) NOT #5 | 1,785 |
| #7 | **Filters: published in the last 5 years** | **1,119** |

**Search without MeSH terms, covering the last 1 year:**

| **Query number** | **Query** | **Items found** |
| --- | --- | --- |
| #8 | "Information storage and retrieval"[Mesh] OR "data repository"[Title/Abstract] OR "data repositories"[Title/Abstract] OR "data base"[Title/abstract] OR "data bases"[Title/Abstract] OR "database"[Title/Abstract] OR "databases"[Title/Abstract] OR "platforms"[Title/Abstract] OR "platform"[Title/Abstract] OR "warehouse"[Title/Abstract] OR "warehouses"[Title/Abstract] | 304,152 |
| #9 | "clinical"[Title/Abstract] OR "medical"[Title/Abstract] OR "biomedical"[Title/Abstract] OR "translational"[Title/Abstract] | 2,862,543 |
| #10 | "omics"[Title/Abstract] OR "genomics"[Title/Abstract] OR "transcriptomics"[Title/Abstract] OR "proteomics"[Title/Abstract] OR "metabolomics"[Title/Abstract] OR "biomarker"[Title/Abstract] OR "biomarkers"[Title/Abstract] OR "molecular"[Title/Abstract] OR "biological"[Title/Abstract] | 1,327,752 |
| #11 | (#1 AND #2 AND #3) | 8,914 |
| #12 | **Filters: published between 2012/09/19 - 2013/10/21** | **1,432** |
|  |  |  |
| #13 | **(#7 OR #12) AND english[Language]** | **2,359** |
